# Remyelination Induced by a DNA Aptamer in a Mouse Model of Multiple Sclerosis

**DOI:** 10.1371/journal.pone.0039595

**Published:** 2012-06-27

**Authors:** Branislav Nastasijevic, Brent R. Wright, John Smestad, Arthur E. Warrington, Moses Rodriguez, L. James Maher

**Affiliations:** 1 Department of Biochemistry and Molecular Biology, Mayo Clinic College of Medicine, Rochester, Minnesota, United States of America; 2 Departments of Neurology and Immunology, Mayo Clinic College of Medicine, Rochester, Minnesota, United States of America; Tokyo Medical and Dental University, Japan

## Abstract

Multiple sclerosis (MS) is a debilitating inflammatory disease of the central nervous system (CNS) characterized by local destruction of the insulating myelin surrounding neuronal axons. With more than 200 million MS patients worldwide, the absence of treatments that prevent progression or induce repair poses a major challenge. Anti-inflammatory therapies have met with limited success only in preventing relapses. Previous screening of human serum samples revealed natural IgM antibodies that bind oligodendrocytes and promote both cell signaling and remyelination of CNS lesions in an MS model involving chronic infection of susceptible mice by Theiler’s encephalomyelitis virus and in the lysolecithin model of focal demyelination. This intriguing result raises the possibility that molecules with binding specificity for oligodendrocytes or myelin components may promote therapeutic remyelination in MS. Because of the size and complexity of IgM antibodies, it is of interest to identify smaller myelin-specific molecules with the ability to promote remyelination *in vivo*. Here we show that a 40-nucleotide single-stranded DNA aptamer selected for affinity to murine myelin shows this property. This aptamer binds multiple myelin components *in vitro*. Peritoneal injection of this aptamer results in distribution to CNS tissues and promotes remyelination of CNS lesions in mice infected by Theiler’s virus. Interestingly, the selected DNA aptamer contains guanosine-rich sequences predicted to induce folding involving guanosine quartet structures. Relative to monoclonal antibodies, DNA aptamers are small, stable, and non-immunogenic, suggesting new possibilities for MS treatment.

## Introduction

MS is a debilitating neurological disease with a prevalence of about 0.1% in the Western world [1]. Though studied for more than 150 years, the causes of this apparent immune-mediated disorder remain unknown. Distinct patterns of MS disease have been discerned [2], and the uneven geographical distribution of MS cases is perplexing. MS is fundamentally an inflammatory disease leading to CNS lesions characterized by the loss of myelin required for electrical insulation of neuronal axons [3], [4]. Resulting symptoms, including fatigue, gait impairment, cognitive impairment, and vision loss, can lead to permanent disability [5]. Hypotheses for MS causation include autoimmune, genetic, environmental, and infectious factors, though no consensus has been reached.

While the origin of MS remains unresolved, therapy and cure present even more urgent challenges. Therapies for relapsing MS include plasma exchange to remove pathogenic immunoglobulins and/or treatment with anti-inflammatory drugs such as glatiramer acetate, β interferon, mitoxantrone, and natalizumab [6]. These approaches are not curative, and are ineffective in some cases [7]. It remains unclear whether curative therapy should be directed against the immune system, or toward repair and rescue of oligodendrocytes and myelin.

A fortuitous observation led to the discovery of antibodies that promote remyelination [8]. In these studies, passive transfer of antisera induced by immunization of mice with myelin was observed to promote remyelination in MS-like lesions induced by chronic infection by Theiler’s encephalomyelitis virus (TMEV), suggesting a therapeutic role for anti-myelin antibodies. Similar results were obtained in the lysolecithin model of focal demyelination [9]. Subsequent screening led to the identification of multiple natural murine and human IgM autoantibodies that bind to live cerebellum and cultured oligodendrocytes and promote remyelination in mice [8]. Target antigens are not known in molecular detail, but the pentavalent character of the IgM antibody is important for activity [10]. A recombinant form of one such IgM antibody, sHIgM22, is in preclinical development. Because some myelin-specific ligands bind oligodendrocytes and promote remyelination in MS lesions, we are seeking alternative agents that are smaller and more robust than IgM monoclonal antibodies. In the present study we applied *in vitro* selection to identify a small single-stranded DNA aptamer with affinity for myelin and the ability to promote remyelination in mice (Fig. S1).

Aptamers are folded, single-stranded nucleic acids with activities that, like folded proteins, depend on their three-dimensional shapes and surface features [11], [12]. Although RNA and DNA aptamers are under investigation in a variety of therapeutic contexts [13], their potential has not been fully explored. Potential advantages of aptamers relative to antibodies include their much smaller size, greater chemical stability, ease of synthesis, and lack of immunogenicity. A fundamental advantage of aptamers is the availability of *in vitro* selection technology wherein cycles of affinity selection and amplification can identify nucleic acids with rare properties from vast random libraries containing 10^14^ or more candidates [14]. Such chemical diversity exceeds that encoded in mammalian immune systems, and the selection process takes place *in vitro*.

## Results and Discussion

**Figure 1:**
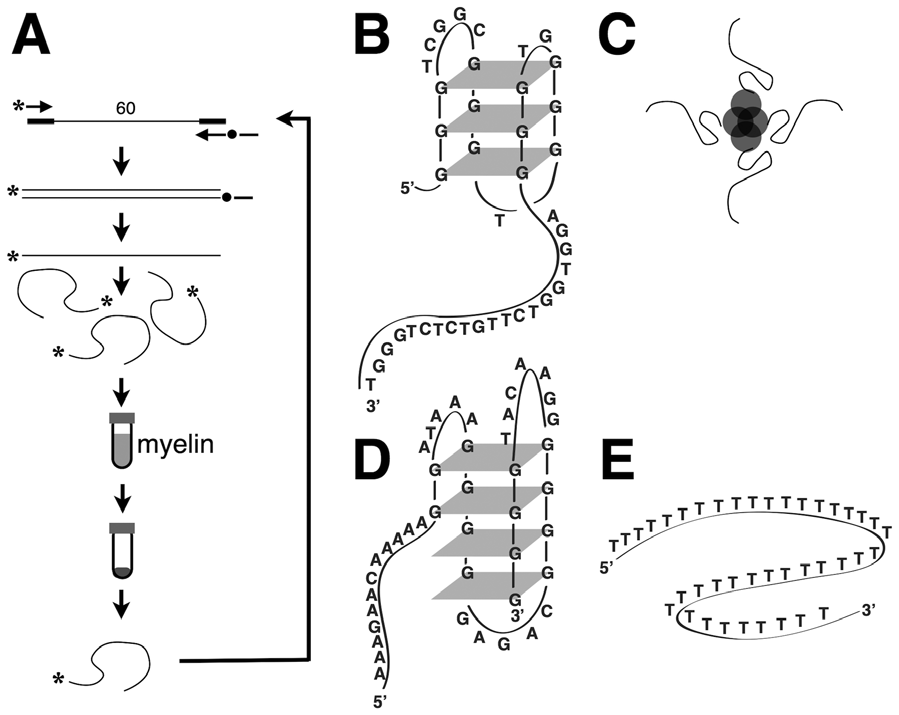
Selection of DNA aptamers specific for components of crude mouse myelin suspension. A. A pool of >10^12^ 100-nucleotide fluorescent (*) single-stranded DNA aptamers containing 60 nucleotides of random sequence is generated by a PCR and mixed with a suspension of crude mouse myelin. Cycles of affinity purification and amplification (Figure S1, S2, S3, S4) yield long myelin-specific DNA aptamers from which active sub-sequences can be derived. B. Deoxyguanosine-rich DNA aptamer 3064 specific for crude mouse myelin, shown folded as a putative intramolecular G-quadruplex. C. Tetravalent complex of 3-biotinylated DNA aptamer (thin lines) with streptavidin (circles). D. Negative control deoxyguanosine-rich DNA aptamer 3060 specific for chelated Nickel ions, shown as putative intramolecular G-quadruplex. E. Negative control oligodeoxythymidylate 3202.

We used *in vitro* selection from a single-stranded DNA library to identify aptamers that bound to a suspension of crude murine myelin (Fig. 1, Fig. S2, S3, S4). The procedure (Fig. 1A) yielded DNA molecules that were sequenced. A resulting anti-myelin aptamer 3064 (Fig. 1B-C) was compared with negative control aptamers 3060 (Fig. 1D) and 3202 (Fig. 1E). Interestingly, and as previously observed for some other DNA aptamers [15]–[17], both aptamer 3064 (specific for myelin) and control aptamer 3060 (selected for affinity to recombinant Myelin Oligodendrocyte Glycoprotein (MOG), but also showing the ability to bind chelated Nickel ions [18]) contain guanosine-rich domains predicted to induce intra- or intermolecular folding through guanosine quartets.

**Figure 2:**
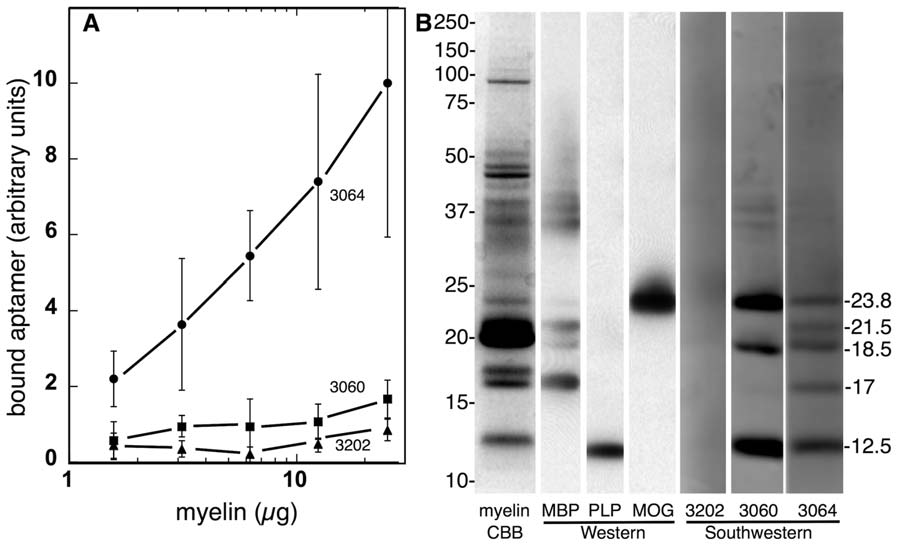
DNA aptamer specificity characterization. A. Binding of fluorescent aptamers 3064 (filled circles), 3060 (filled squares) and 3202 (filled triangles) to crude mouse myelin proteins detected by sedimentation of the insoluble myelin fraction. Mean and standard deviation are shown for three repeats. B. Coomassie staining of crude myelin proteins (myelin; CBB) separated by SDS-polyacrylamide gel electrophoresis and results of western and southwestern blotting with the indicated antibodies or fluorescent aptamers. Mobilities of molecular weight standards are indicated at left and apparent molecular weights of aptamer-reactive proteins are indicated at right.

We analyzed the specificity of anti-myelin DNA aptamer 3064 and controls 3060 and 3202 by assessing the binding of fluorescent aptamers to crude myelin protein suspension using centrifugal sedimentation to recover bound aptamers (Fig. 2A). Myelin-specific aptamer 3064 showed strong myelin-dependent binding while aptamers 3060 and 3202 did not. Because the myelin preparation is a crude mixture of proteins and lipids, these results cannot provide quantitative affinity estimates. To further assess specificity, crude myelin proteins were separated by SDS-polyacrylamide gel electrophoresis (Fig. 2B) and stained with Coomassie dye (myelin) or the proteins blotted to polyvinylidene *(*PVDF) membrane followed by probing with antibodies to myelin basic protein (MBP), proteolipid protein (PLP), or MOG in western blots, or probing with fluorescent aptamers 3202, 3060 or 3064 in southwestern blots. Interestingly, whereas control aptamer 3202 showed no binding to myelin proteins, guanosine-rich aptamers 3060 and 3064 bound specifically to certain proteins (Fig. 2B). In particular, control aptamer 3060 and myelin-specific aptamer 3064 both bound to three myelin proteins with apparent molecular weights of 12.5 kDa, 18.5 kDa, and 23.8 kDa. Based on western analysis and mass spectrometry of tryptic peptides these proteins are PLP, the 18.5 kDa isoform of MBP, and MOG, respectively. Importantly, myelin-specific aptamer 3064 uniquely bound to myelin proteins with apparent molecular weights of 17 kDa and 21.5 kDa, identified as MBP isoforms containing sequences encoded by MBP exon 2 [19]. Interestingly, it has previously been suggested that immune reagents targeting exon 2 sequences can stimulate remyelination [20].

It is interesting to note that DNA aptamer 3064 is observed to bind preferentially to an intact myelin suspension (Fig. 2A), while both aptamers 3060 and 3064 show affinity for certain myelin proteins after extraction of lipids and immobilization (Fig. 2B). As discussed above, these results suggest that MBP sequences encoded in exon 2 are targeted by aptamer 3064 and these sequences are preferentially accessible in crude myelin suspensions. Extraction of proteins for Southwestern blotting reveals other aptamer binding sites that may not be accessible in intact myelin.

**Figure 3:**
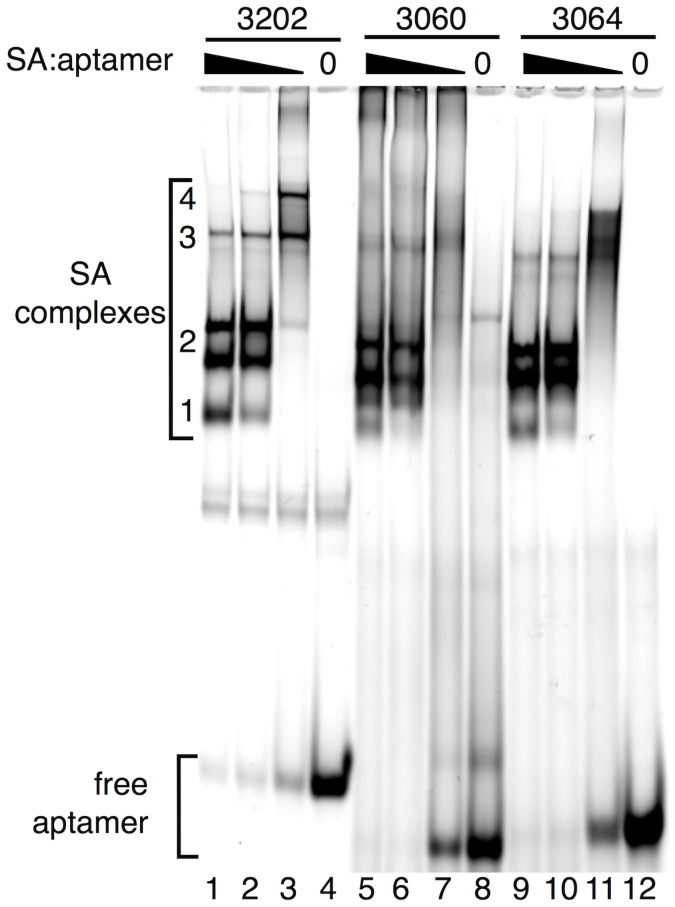
Formation of 3′-biotinylated DNA aptamer multimers by incubation with streptavidin. The indicated 5′-fluoresceinated, 3′-biotinylated DNA aptamers (lanes 4, 8, 12) were folded and then incubated with different amounts of streptavidin to produce multimeric aptamer complexes. Aptamer:streptavidin concentration ratios were 1∶1 (lanes 1, 5, 9), 4∶1 (lanes 2, 6, 10), and 20∶1 (lanes 3, 7, 11). Mobilities of aptamer monomer and complexes containing one to four aptamers (1–4; see Fig. 1C) are indicated. Note that complexes with two bound aptamers display two distinct mobilities due to cis vs. trans binding arrangements on the streptavidin tetramer.

The binding properties of anti-myelin DNA aptamer 3064 are similar to certain natural human IgM autoantibodies that promote remyelination in the murine TMEV model of MS [8]. We therefore tested intraperitoneal injection of anti-myelin DNA aptamer 3064 compared to negative control aptamers 3060 and 3202. Aptamers were prepared as 3′-biotin conjugates and incubated in a 4∶1 molar ratio (Fig. 3) with tetrameric streptavidin to create conjugates with enhanced stability and biodistribution [21] mimicking polyvalent antibodies. As shown in Fig. 3 (lanes 2, 6, 10), 4∶1 aptamer:streptavidin incubation under these conditions converts all monomeric aptamers to streptavidin complexes, predominantly dimers.

**Figure 4:**
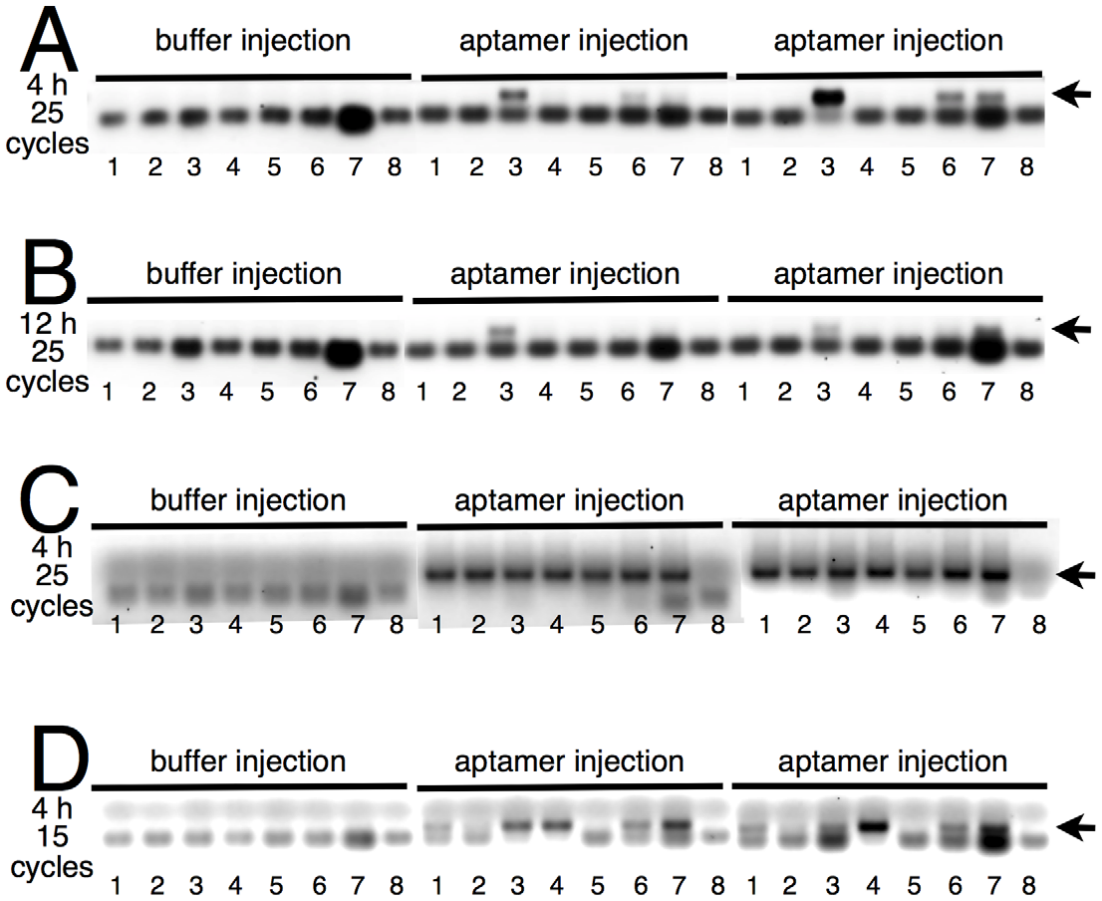
Tissue distribution of DNA aptamer 3064 conjugated to biotin 4 h (A, C, D) or 12 h (B) after i.p. injection into healthy FVB mice (A, B) or TMEV-infected SJL/J mice (C, D). Control animals (“buffer injection” group in each panel) were injected with the equivalent buffer solution lacking aptamer conjugate. Conjugates were extracted from duplicate mice (middle and right group in each panel) and detected by the indicated number of PCR cycles as described in Methods. Each lane reflects PCR products amplified from extract representing 8.25 mg original tissue wet weight. Arrows indicate position of 120-bp aptamer-specific PCR product. Numbering: 1, heart; 2, spinal cord; 3, spleen; 4, lung; 5, brain; 6, kidney; 7, liver; 8, water control. A nonspecific prominent PCR product (higher mobility than the aptamer-specific product) results from primer-primer interactions.

To explore the biodistribution of DNA aptamer 3′-biotin conjugates, healthy FVB mice (Fig. 4 A, B) or TMEV-infected SJL/J mice (Fig. 4 C, D) were treated by a single intraperitoneal (i.p.) injection of 3064 aptamer conjugate (500 µl of 1 µM conjugate solution; 0.5 nmol) or buffer alone and various tissues were harvested 4 h or 12 h after injection. A PCR-based assay was developed to detect the 3064 aptamer in tissue extracts. As shown in Fig. 4, no aptamer signal was detected in animals injected with buffer. For healthy FVB mice, aptamer was readily detected in spleen, and to a lesser extent in kidney and liver, 4 h and 12 h after injection. Aptamer could not be detected in heart, spinal cord, lung, or brain under these conditions in healthy animals. Remarkably, aptamer biodistribution was much enhanced in TMEV-infected mice. Four hours after i.p. injection, DNA aptamer could readily be detected in all tested tissues, including brain and spinal cord (Fig. 4C). Detection after only 15 PCR cycles indicated highest aptamer levels in organs of the abdomen and thorax (Fig. 4D).

**Figure 5:**
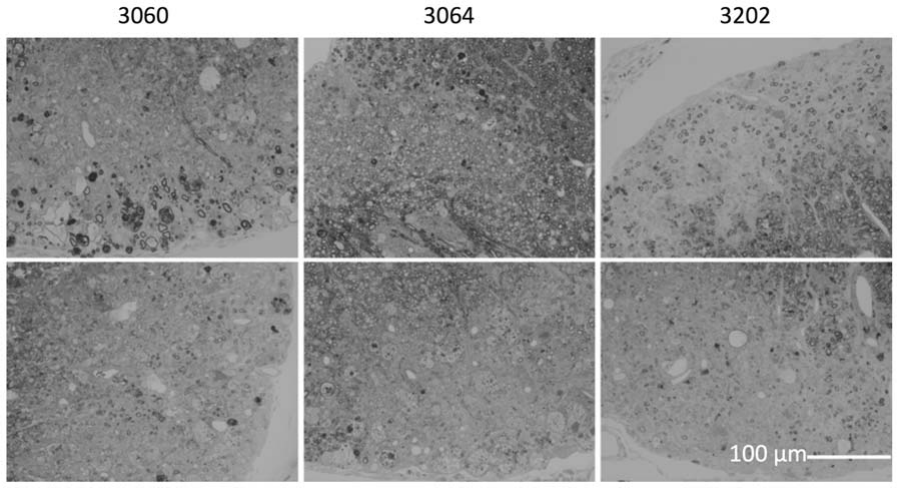
Light photomicrographs demonstrating examples of TMEV-mediated spinal cord demyelination (lower panels) and remyelination (upper panels) in mice treated with the indicated DNA aptamers. Scale bar in lower right panel is 100 micrometers. After blinded micrograph review of specimens from control and aptamer-treated animals the percent of spinal cord quadrants showing demyelination or remyelination was determined as reported in Table 1.

To study aptamer conjugate effects on CNS remyelination, TMEV-infected SJL/J mice were treated by i.p. injection of various aptamer conjugates (500 µl of 1 µM conjugate solution; 0.5 nmol) twice per week for a total of 10 doses starting 27 weeks after Theiler’s virus infection. Central nervous system pathology was then assessed 5 weeks after completion of aptamer injections. Results are shown in Fig. 5 and Table 1. Spinal cords from all mice in the study contained areas of chronic demyelination. Infiltrating macrophages were present in several lesions. Remyelination is characterized by densely packed thin myelin sheaths in relation to axon diameter. In mice treated with anti-myelin aptamer 3064 more areas of dense remyelinated axons were found (Fig. 5). For example, the top middle panel of Fig. 5 shows an area of almost complete remyelination mediated by oligodendrocytes located in the dorsal white matter column of the spinal cord of a mouse treated with anti-myelin aptamer 3064. In mice treated with control aptamers 3060 and 3202 dorsal column spinal cords lesions contained fewer remyelinated axons. As shown in Table 1, anti-myelin DNA aptamer 3064 induced remyelination in 35% of experimental CNS lesions, compared to 4% and 9% remyelination after treatment with negative control aptamers 3202 and 3060, respectively. These statistically significant results indicate selective enhancement of remyelination *in vivo* by anti-myelin DNA aptamer treatment. Interestingly, animals injected with streptavidin and non-biotinylated aptamers 3064 or 3060 did not show remyelination. This result suggests that aptamer 3′ modification is important for protection for nuclease attack and/or streptavidin binding to form multivalent conjugates.

**Table 1:** Remyelination *in vivo* after DNA aptamer treatment^1^.

aptamer	biotin	streptavidin	N	Demyelination	remyelination
(specificity)					
3064	+	+	10	41.1±6.4	34.9±6.1
(myelin)					
3060	+	+	7	38.2±8.7	8.8±4.5
(Nickel)					
3202	+	+	8	51.8±26.4	4.2±2.3
(dT_40_control)					
3064	–	+	9	42.0±5.8	8.5±3.4
(myelin)					
3060	–	+	8	50.0±4.6	10.3±4.4
(Nickel)					

1 Table indicates lesion status, by animal, after blinded review of neuropathology. N: number of mice. Data express percentage of spinal cord quadrants showing at least 75% of the lesion was remyelinated (mean +/− SEM). Statistical comparisons: P<0.002 remyelination - (ANOVA on Ranks). Dunn’s comparison to 3202 (control) showed statistical difference (p<0.05) against 3064 with biotin/streptavidin. There was no statistically significant difference in remyelination between 3202 control and 3064 without biotin.

This aptamer-induced remyelination can be compared with prior effects obtained using much larger and more labile human IgM autoantibodies. In the latter case, a single dose (0.6 nmol) IgM antibody promoted ∼60% remyelination vs. 15% observed for negative control [22]. The present result raises the possibility that the observed remyelination activity of anti-myelin DNA aptamers may also reflect direct interactions with lesions, though this remains to be demonstrated. It has previously been shown that the abilities of certain natural IgM autoantibodies to stimulate cell signaling and remyelination depend on the multivalent character of the antibody structure [10]. It is noteworthy that the formulation of biotinylated DNA aptamers with streptavidin was intended to promote the formation of streptavidin-linked multivalent aptamer conjugates based on intramolecular aptamer folding (Fig. 1B).

A number of important mechanistic issues remain to be better understood for both antibodies and DNA aptamers that appear to promote remyelination. These include the question of whether the agents reach the demyelinated regions of the CNS and function directly or indirectly, their actual target epitope(s), and their detailed dose-response profiles. Evidence of spleen uptake in healthy mice (Fig. 4) suggests the potential for a systemic immune modulation mechanism, and direct effects on the CNS are possible in TMEV-infected animals where the blood brain barrier is apparently compromised by inflammation. Eventually it will also be important to measure the functional impact of aptamer-promoted remyelination in both mouse models and human trials.

The molecular mass of the anti-myelin DNA aptamer tested here is ∼13,000. If fully tetramerized with streptavidin (mass ∼52,800) the resulting complex with mass ∼104,800 is still about 10-fold smaller than the IgM antibodies we have previously shown to promote remyelination. IgM antibodies are more difficult to manufacture, are more likely to be immunogenic, but have been shown to cross the blood-brain barrier. DNA aptamers can be prepared by chemical synthesis, but future studies will be required to assess the immunogenicity and tissue distribution of the aptamer conjugates shown here to promote remyelination. These considerations motivate further exploration of DNA aptamer reagents for MS treatment.

## Materials and Methods

### Preparation of Crude Murine Myelin

CNS tissue from strain SJL mice (5 g) is homogenized in 0.32 M sucrose containing 2 mM EGTA (pH 7.5) using a tissue grinder followed by a Dounce homogenizer to yield a final volume of 100 ml. 17 ml of homogenate is layered onto 3 ml of 0.85 M sucrose containing 2 mM EGTA and subjected to centrifugation at 28,000 rpm for 1 h at 4°C. Material is collected from the interface and homogenized in a total volume of 240 ml solution containing 2 mM EGTA. After centrifugation, the pellet is homogenized in 5 ml of a solution containing 10 mM EGTA, the volume brought to 400 ml in 10 mM EGTA, and the solution stirred for 15 min at 4°C. After centrifugation for 15 min at 10,000 rpm, homogenization and centrifugation is repeated. The resulting pellet is homogenized in 100 ml of solution containing 0.85 M sucrose and 2 mM EGTA. The homogenate is overlayed with 3 ml solution containing 0.32 M sucrose and 2 mM EGTA, subjected to centrifugation at 28,000 rpm for 90 min. After repeated homogenization and washing the myelin is isolated from the 0.32 M/0.75 M interface of a discontinuous sucrose gradient, washed with distilled water, and resuspended in 50 mM Tris-HCl containing 2 mM EGTA.

### *In vitro* Selection of DNA Aptamers

The initial round of selection employed 2.5 nmol (∼1×10^15^ molecules) of random oligonucleotide library LJM-2772. Oligonucleotides were heated to 90°C for 1 min in PBS containing 1 mM MgCl_2_, placed on ice for 15 min, and then incubated for 8 min at room temperature to allow folding. 200 µL mouse myelin suspension (10 µg) was pelleted by centrifugation for 5 min at 6500 rpm (microcentrifuge). The pellet was washed twice by resuspension in 500 µL binding buffer (20 mM Tris-HCl, pH 7.6, 10 mM NaCl, 0.5 mM KCl). The DNA library (5 µM in round 1, 300 nM in subsequent rounds) was then incubated for 30 min with gentle agitation in a 500 µL binding reaction with 10 µg myelin suspension in 500 µL binding buffer. The suspension and bound aptamers was washed twice with 1 mL binding buffer by 6500 rpm centrifugation. To the pellet was added 400 µL 2× PK buffer (300 mM NaCl, 2.5 mM EDTA, 2% SDS), followed by agitation, and extraction with 400 µL phenol:chloroform (1∶1, v:v). DNA was precipitated from the aqueous phase by addition of ethanol. A portion of the recovered DNA was amplified by PCR to establish the optimal number of amplification cycles. After the first round, PCR was performed with a fluorescein-labeled primer, allowing quantitation of library recovery by fluorescence spectroscopy. The upper primer sequence was 5′-F-ATAC_2_AGCT_2_AT_2_CA_2_T_2_ (F indicates fluorescein). The lower primer sequence was 5′-A_20_-X_2_-AGAT_2_GCACT_2_ACTATCT (X indicates GLEN Research spacer phosphoramidite 10-1909). PCR reactions (100 µL) employed Taq DNA polymerase, primers at 10 µM final concentration, and incubation for 5 min at 94°C, followed by cycles of 30 s at 94°C, 30 s at 47°C, and 30 s at 72°C. A second aliquot of recovered DNA was then amplified for the optimum number of cycles to prepare aptamer for the next selection round. Single-stranded fluorescent aptamer was obtained by precipitation of PCR reactions from ethanol, followed by denaturing polyacrylamide gel electrophoresis. The fluorescent DNA band was cut from the gel, diced, and eluted in TE buffer at 37°C for 2–12 h, followed by precipitation from ethanol and quantitation by UV spectrometry. After 11 selection cycles, PCR was performed and the resulting duplex DNA was ligated into the pGEM-Teasy cloning vector (Promega, Madison, WI), cloned, and sequenced.

### Aptamer Specificity Characterization

To promote intramolecular folding, aptamer stock solutions (5 µM) in PBS were heated to 90°C and MgCl_2_ was then added to a final concentration of 1 mM and solutions were allowed to cool room temperature. Myelin stock was diluted in PBS and sonicated on ice. Fluorescent folded aptamers were added to different amounts of myelin suspension and incubated at 37° C for 3 h. Insoluble material with bound aptamers was recovered by 30 s centrifugation in the microcentrifuge. The pellet was washed with 100 µL PBS and again recovered by centrifugation. After phenol extraction and ethanol precipitation fluorescent aptamers were quantitated in black plastic 96-well plates using a Typhoon Fluorescent Imaging system (GE). For Southwestern blotting, 15 µg crude myelin protein was separated in each lane of a 10% Bis-Tris SDS polyacrylamide gel in MES buffer. After electrophoresis, duplicate lanes were either stained with Coomassie blue dye or transferred to PVDF membrane by electroblotting. Western blotting was performed by standard methods using antibodies with the indicated specificities. For southwestern blots, membranes were blocked for 30 min at 37° C in TBST buffer containing with 1% BSA, 10% non-fat dry milk, 2 mg/mL sonicated and heat-denatured salmon testis DNA, and Tween 20 detergent. Folded fluorescent aptamers (5 µM final concentration) were then added in PBS containing 1 mM MgCl_2_ and incubated with PVDF membranes for 14 h followed by washing in TBST buffer containing Tween 20 detergent and then washing with 0.5× TBE buffer. Fluorescein fluorescence was then detected on membranes using the Typhoon Fluorescent Imaging system (GE). Tryptic peptide mass fingerprinting was performed in the Mayo Clinic Proteomics Center.

### Mouse Model

All studies conformed to Mayo Clinic and National Institutes of Health animal use guidelines and were reviewed and approved by the Mayo Clinic Institutional Animal Care and Use Committee as protocol A29509. Eight-week-old female SJL/J mice (Jackson Laboratories, Bar Harbor, ME) received a single intracerebral injection of 2 × 10^5^ plaque-forming units of the Daniel’s strain of Theiler’s Myeloencephilitus Virus (TMEV) in Dulbecco’s phosphate buffered saline (DPBS; 10 µL). The resulting encephalitic-like infection resulting in greater than 98% incidence of demyelination with increasing neurologic deficits progressing over several months [23]. Animals used for remyelination studies in this report were chronically demyelinated by 6 months post infection with clear neurologic deficits. To assemble treatment groups in cages of five mice each, all mice to be treated were combined in a large container and then distributed equally based on the level of disability. The extent of mouse disability was determined by examination of the mouse coat color, a reflection of the ability to self-groom, gait and the ability to right when placed on the dorsal side.

### Aptamer Treatment

DNA oligonucleotides LJM-3064b (40 nt), LJM-3060b (43 nt) and LJM-3202b (40 nt) were synthesized DMT-off at 1 µmol scale using 3′ biotinTEG control pore glass support (Glen Research 20-2955). Oligonucleotides were cleaved from the support and deprotected in hot ammonia, then dried and purified by reverse phase HPLC and sterilized by precipitation from ethanol. Groups of mice received 500-µL intraperitoneal (i.p.) injections of the 3′ biotin conjugated aptamer (1 µM) combined with streptavidin (0.25 µM) in Calcium-free D-PBS (Invitrogen) supplemented with magnesium chloride (1 mM). Injections were twice per week for 5 weeks. Briefly, sterile aptamer solution (1 µM) in Calcium-free D-PBS supplemented with MgCl_2_ (1 mM) was heated to 90° C for 1 min, placed on ice for 15 min, and then incubated for 8 min at room temperature to allow aptamer folding. Streptavidin stock solution (Jackson Immune Research; 1 mg/mL; 18 µM in Calcium-free D-PBS) was added to a final concentration of 0.25 µM and incubated with gentle agitation for 30 min at 37° C immediately prior to intraperitoneal injection into mice. The final aptamer injection solution (500 µL) contained streptavidin: 13.8 µg/mL (0.25 µM), aptamer-3′-Biotin: 13.4 (12.7–14.2) µg/mL (1.2–1.5 µM) in Calcium-free D-PBS supplemented with MgCl_2_ (1 mM). Each treatment therefore consisted of 6.9 µg (125 pmol) streptavidin, 6.7 µg (500 pmol) aptamer-3′-biotin, and 47.6 µg (500 µmol) MgCl_2_.

### Aptamer Biodistribution

Healthy FVB mice (38 d) or TMEV-infected SJL/J mice (6 months post infection) were used for aptamer biodistribution studies. DNA aptamer-streptavidin conjugates were extracted 4 h or 12 h after a single i.p. injection of 500 µL 1 µM biotinylated DNA aptamer 3064 prepared as low-order conjugates conjugate with streptavidin in Calcium-free D-PBS containing 1 mM MgCl_2_. Control animals were injected with the equivalent solution lacking aptamer conjugate. Animals were killed by sedation with isoflurane vapors followed by IP injection of 0.1 mL sodium barbital (constituting a lethal dose). Tissues (80–150 mg/organ)were harvested and homogenized in Qiagen plasmid preparation buffer P1 (300 µL), followed by addition of Qiagen plasmid preparation buffer P2 (300 µL), and then precipitation of genomic DNA, proteins and cellular debris by addition of Qiagen plasmid preparation buffer P3 (300 µL). After centrifugation, the clarified supernatant was treated with proteinase K (0.1 mg/ml final concentration) overnight at 55° and nucleic acids precipitated by addition of an equal volume of isopropanol and centrifugation. DNA aptamer 3064 was detected by PCR using primers:

LJM-4556 (5′-CTAGACTAGA_2_GCTGAGCTGCTAGACTAGA_2_GCTGAGCTG_4_TCG_2_CG_3_TG_3_) and LJM-4557 (5′- ACGT_2_ACGT_2_ATGACATGACACGT_2_ACGT_2_ATGACATGACAC_3_AGAGACA_2_GAC_2_AC) and Failsafe Kit (Epicentre, Madison, WI) condition J or L with the first five cycles annealed at 40° C and subsequent cycles annealed at 60° C. An aptamer-specific PCR product of 120 bp is generated from 40-nucleotide aptamer 3064.

### Spinal Cord Morphometry

Mice were euthanized with sodium pentobarbital and perfused intracardially with Trump’s fixative (phosphate-buffered 4% formaldehyde/1% glutaraldehyde, pH 7.4). Spinal cords were removed, cut into 1 mm blocks and every third block fixed and stained with osmium tetroxide and embedded in araldite plastic (Polysciences, Warrington, PA). One-micrometer-thick cross-sections were cut from each block, mounted onto glass slides, and stained with 4% paraphenylene-diamine to visualize myelin [24]. 10–12 cross-sections represent samples from the cervical, thoracic, lumbar, and sacral spinal cord. Neuropathology to characterize extent of lesion remyelination was performed according to a blinded protocol. Three investigators (MF, BW and AW) examined the sections independently without knowledge of treatment groups. For blinded grading, each spinal cord section was divided visually into four quadrants based on morphological symmetry and examined by bright field microscopy at 100× and 200× total magnification using an Olympus Provis microscope. Demyelinated areas were characterized by denuded axons and inflammatory cell infiltrates. Demyelinated areas with remyelination were characterized by thin myelin sheaths compared with the thicker, intact myelin sheaths. The spinal cord white matter was scored as normal, demyelinated with no remyelination, or demyelinated with remyelination [22]. Partial quadrants were excluded. Lesions were judged to be remyelinated when the lesion was 75–100% repaired. Remyelinated lesions below this threshold were scored as negative. Data were not assembled into treatment groups until all slides in a given study were graded. Demyelination for each mouse was calculated as a percentage based on the number of spinal cord quadrants with demyelination, which includes those quadrants with demyelination and repair, divided by the total number of quadrants scored. Remyelination for each mouse was calculated as a percentage based on the number of demyelinated quadrants above threshold remyelination divided by the number of quadrants with demyelination. Data for percentage spinal cord demyelination and remyelination were compared across groups using one-way ANOVA on ranks. When a significant difference (P<0.05) was identified, a pairwise comparison of aptamer-treated groups with the aptamer-control and untreated control groups was performed. Statistical analysis and plots were performed using SigmaStat and SigmaPlot.

## Supporting Information

Figure S1 **Schematic of approach and results.** A vast random pool of ∼10^15^ single-stranded DNA molecules is generated. DNA molecules (aptamers) are selected for binding to components of a crude mouse myelin suspension. After cloning and identification of the DNA subsequence important for myelin binding, DNA aptamers are injected into the peritoneal cavities of mice with demyelinating CNS lesions induced by Theiler’s encephalomyelitis virus infection. Immunopathology is monitored 7–9 months after infection to detect enhanced CNS remyelination.
(TIFF)

Figure S2 **Chemical modifications of DNA aptamers relevant to this work.** A. 5′-fluorescein modification present for *in vitro* selection and binding studies. B. 3′ biotin modification present in aptamers used for *in vivo* injection.
(TIFF)

Figure S3 **Results of** ***in vitro*** **selection of DNA aptamer pools over 10–11 rounds of selection and amplification.** Targets were myelin oligodendrocyte glycoprotein (MOG) immobilized on Ni-NTA magnetic beads (filled circles) or suspension of crude mouse myelin in buffer (open circles). MOG selections gave rise to aptamer 3060 (selective for chelated Nickel beads). Myelin selections gave rise to aptamer 3064.
(TIFF)

Figure S4 **Sequences of anti-myelin DNA aptamers and derived sub-sequences.** A. Initial sequences of DNA aptamers after cloning. Sequences derived from random regions are in black. Fixed sequences for PCR primer binding are in blue. The top three identical sequences correspond to aptamer 3028. B. Derivation of aptamer sub-sequences from anti-myelin aptamer 3028. Aptamer 3064 was selected for further testing in this work.
(TIFF)
