# Supplementary figures and images for: Remyelination Induced by a DNA Aptamer in a Mouse Model of Multiple Sclerosis
Source: PLoS One. 2012 Jun 27;7(6):e39595. doi: 10.1371/journal.pone.0039595 (PMC3384608; doi:10.1371/journal.pone.0039595)

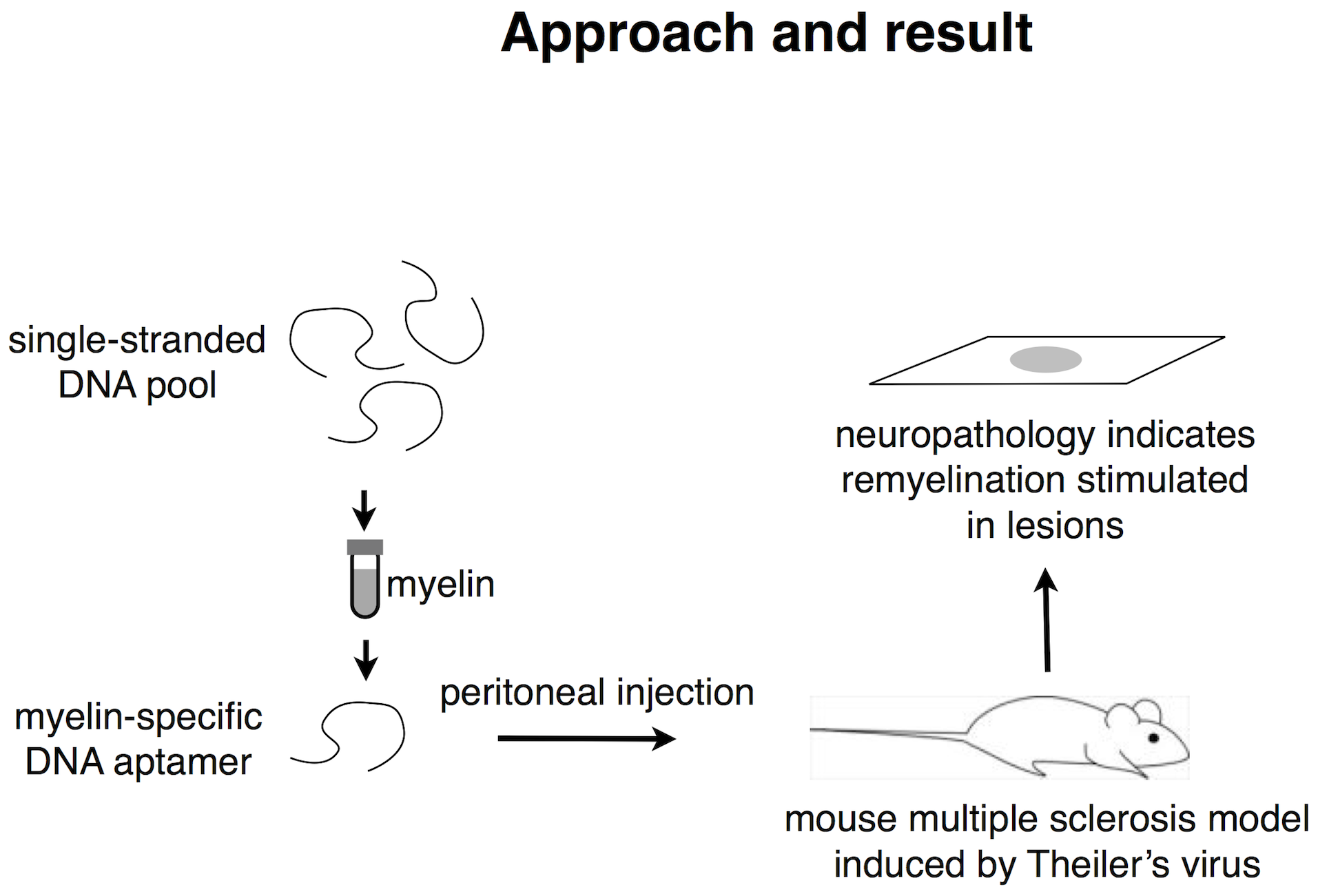

Supplement: Figure S1 — Schematic of approach and results. A vast random pool of ∼1015 single-stranded DNA molecules is generated. DNA molecules (aptamers) are selected for binding to components of a crude mouse myelin suspension. After cloning and identification of the DNA subsequence important for myelin binding, DNA aptamers are injected into the peritoneal cavities of mice with demyelinating CNS lesions induced by Theiler’s encephalomyelitis virus infection. Immunopathology is monitored 7–9 months after infection to detect enhanced CNS remyelination. (TIFF) [file pone.0039595.s001.tif]

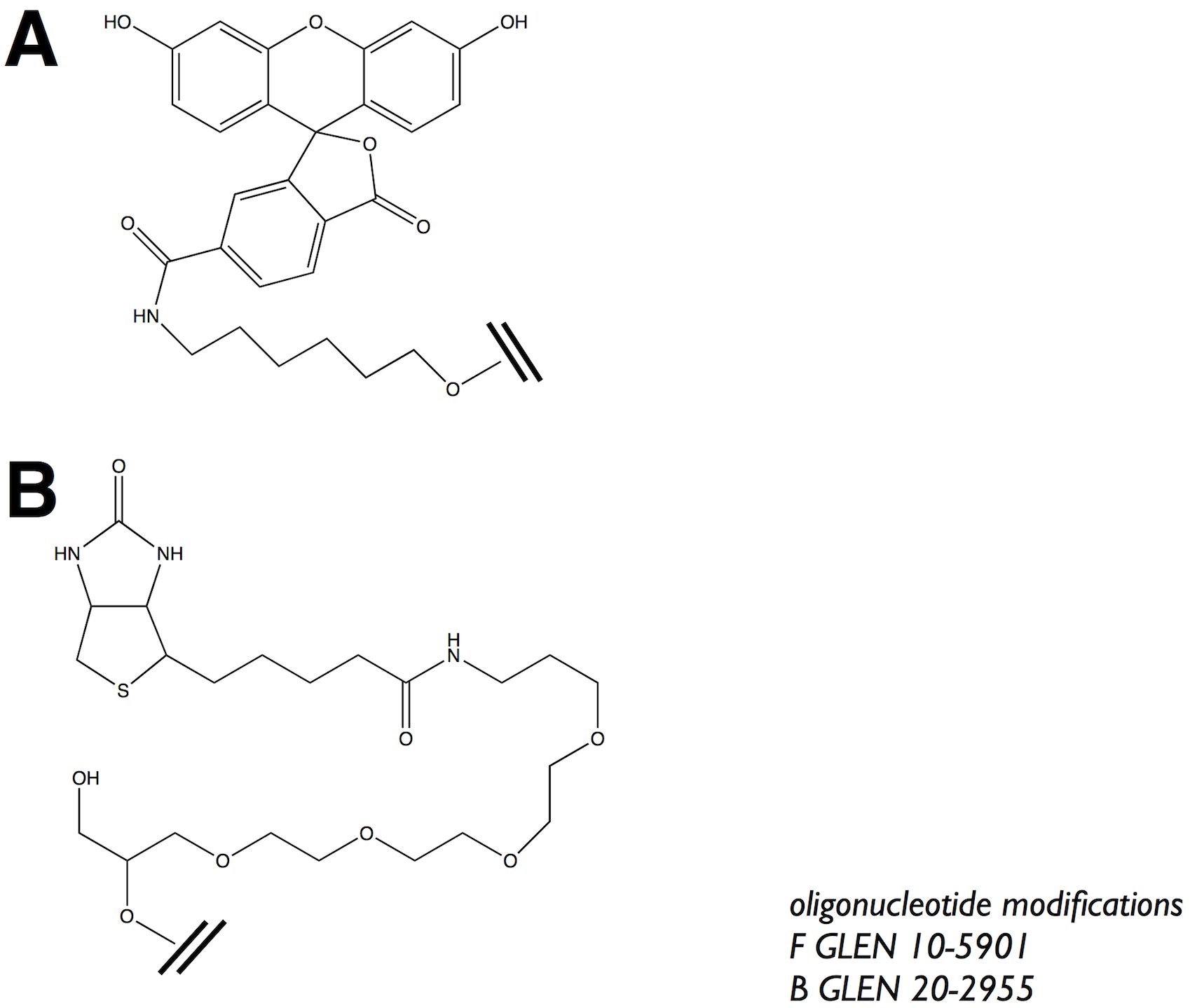

Supplement: Figure S2 — Chemical modifications of DNA aptamers relevant to this work. A. 5′-fluorescein modification present for in vitro selection and binding studies. B. 3′ biotin modification present in aptamers used for in vivo injection. (TIFF) [file pone.0039595.s002.tif]

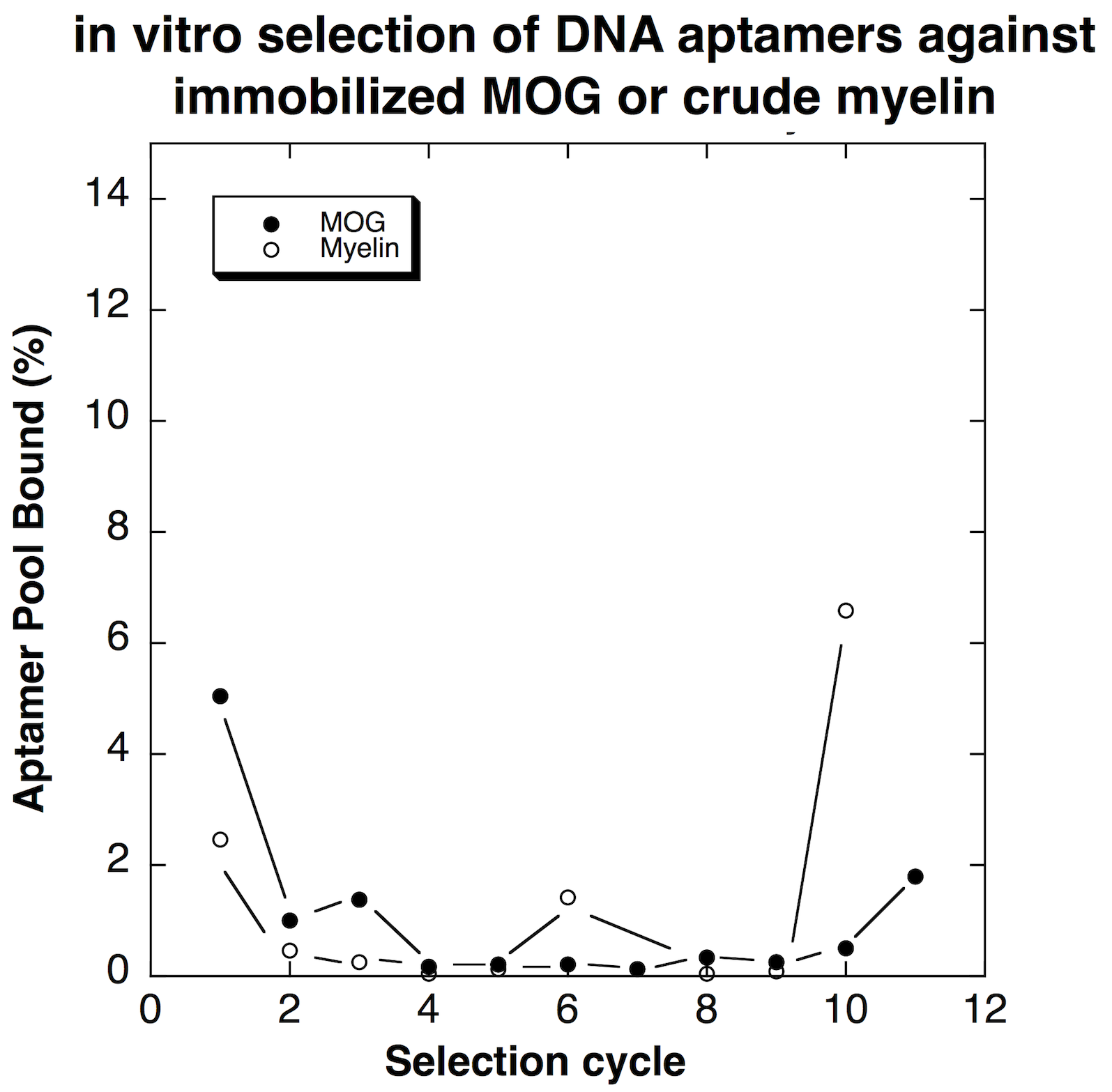

Supplement: Figure S3 — Results of in vitro selection of DNA aptamer pools over 10–11 rounds of selection and amplification. Targets were myelin oligodendrocyte glycoprotein (MOG) immobilized on Ni-NTA magnetic beads (filled circles) or suspension of crude mouse myelin in buffer (open circles). MOG selections gave rise to aptamer 3060 (selective for chelated Nickel beads). Myelin selections gave rise to aptamer 3064. (TIFF) [file pone.0039595.s003.tif]

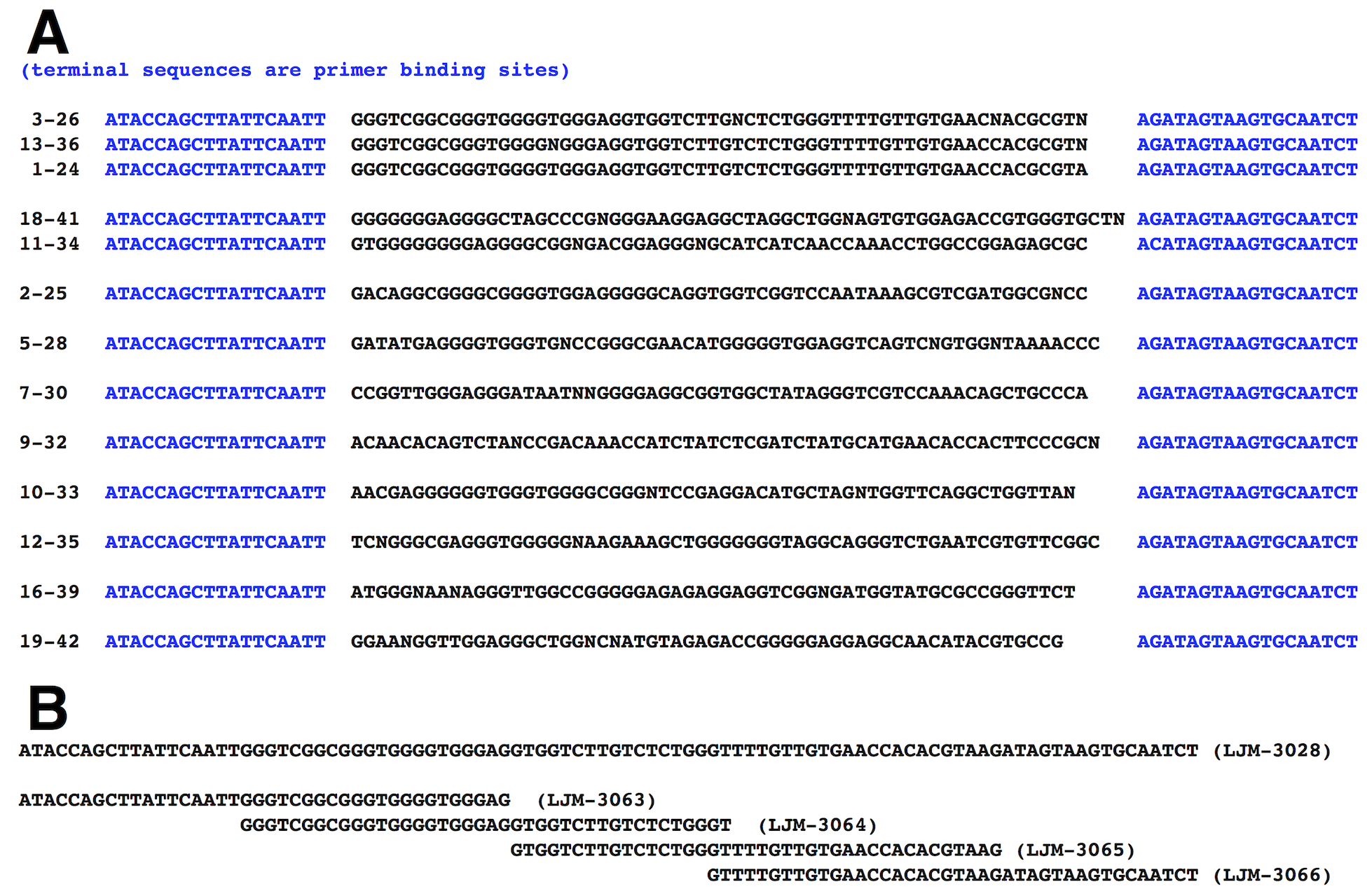

Supplement: Figure S4 — Sequences of anti-myelin DNA aptamers and derived sub-sequences. A. Initial sequences of DNA aptamers after cloning. Sequences derived from random regions are in black. Fixed sequences for PCR primer binding are in blue. The top three identical sequences correspond to aptamer 3028. B. Derivation of aptamer sub-sequences from anti-myelin aptamer 3028. Aptamer 3064 was selected for further testing in this work. (TIFF) [file pone.0039595.s004.tif]
